# The innate face of Giant Cell Arteritis: Insight into cellular and molecular innate immunity pathways to unravel new possible biomarkers of disease

**DOI:** 10.3389/fmmed.2022.933161

**Published:** 2022-08-04

**Authors:** Chiara Rizzo, Lidia La Barbera, Giuseppe Miceli, Antonino Tuttolomondo, Giuliana Guggino

**Affiliations:** ^1^ Department of Health Promotion, Mother and Child Care, Internal Medicine and Medical Specialties, Rheumatology Section, University of Palermo, Palermo, Italy; ^2^ Department of Health Promotion, Mother and Child Care, Internal Medicine and Medical Specialties, Unit of Internal Medicine and Stroke Care, University of Palermo, Palermo, Italy

**Keywords:** giant cell arteritis, innate immunity, inflammation, biomarkers, cytokines, growth factors, reactive oxygen species, endothelial damage

## Abstract

Giant cell arteritis (GCA) is an inflammatory chronic disease mainly occurring in elderly individuals. The pathogenesis of GCA is still far from being completely elucidated. However, in susceptible arteries, an aberrant immune system activation drives the occurrence of vascular remodeling which is mainly characterized by intimal hyperplasia and luminal obstruction. Vascular damage leads to ischemic manifestations involving extra-cranial branches of carotid arteries, mostly temporal arteries, and aorta. Classically, GCA was considered a pathological process resulting from the interaction between an unknown environmental trigger, such as an infectious agent, with local dendritic cells (DCs), activated CD4 T cells and effector macrophages. In the last years, the complexity of GCA has been underlined by robust evidence suggesting that several cell subsets belonging to the innate immunity can contribute to disease development and progression. Specifically, a role in driving tissue damage and adaptive immunity activation was described for dendritic cells (DCs), monocytes and macrophages, mast cells, neutrophils and wall components, such as endothelial cells (ECs) and vascular smooth muscle cells (VSMCs). In this regard, molecular pathways related to cytokines, chemokines, growth factors, vasoactive molecules and reactive oxygen species may contribute to the inflammatory process underlying GCA. Altogether, innate cellular and molecular pathways may clarify many pathogenetic aspects of the disease, paving the way for the identification of new biomarkers and for the development of new treatment targets for GCA. This review aims to deeply dissect past and new evidence on the innate immunological disruption behind GCA providing a comprehensive description of disease development from the innate perspective.

## Introduction

GCA is a granulomatous vasculitis that affects medium- and large-sized vessels in adults older than 50 years of age (Salvarani et al., 2008). GCA encompasses a broad phenotypic spectrum that includes cranial GCA (C-GCA) and large-vessel GCA (LV-GCA), with or without overlapping polymyalgia rheumatica (PMR). C-GCA involves extracranial branches of the carotid artery, preferentially the temporal artery, leading to symptoms such as new onset headache, jaw claudication, scalp tenderness and vision loss. LV-GCA involves the aorta and its proximal branches, including axillary and subclavian arteries, and is characterized by systemic symptoms, aortitis and limb claudication (Weyand and Goronzy, 2003; Dejaco et al., 2017; de Boysson et al., 2018). Vascular inflammation and remodeling can cause arterial steno-occlusion with consequent ischemic manifestations or aortic aneurysms and dissection. GCA is a potential life-threatening disease due to the wide range of complications, coupled with high mortality and great risk of relapse (Pugh et al., 2022). Therefore, an early diagnosis, a prompt therapeutic approach and a strict follow up are key elements in GCA patient workup.

Accordingly, ongoing researches are focused on the evaluation of new potential biomarkers of disease activity and treatment response (Tombetti et al., 2021). Standard routine monitoring of disease activity includes assessment of clinical manifestations, inflammatory markers, primarily erythrocyte sedimentation rate (ESR) and C-reactive protein (PCR) levels, and imaging techniques (Schmidt, 2018; Hellmich et al., 2020). All the mentioned parameters cannot mirror the complexity and heterogeneity of inflammatory vascular disease. Then, accurate rating of vascular disease activity and evaluation of relapse risk are still challenging. To overcome this significant unmet need several serological biomarkers for the diagnosis and follow up of GCA are under investigation. However, to date, no reliable disease-specific biomarkers are available. Serum biomarkers candidates include ESR, CRP, serum amyloid A (SAA) and interleukin (IL)-6 (Burja et al., 2019; O'Neill et al., 2015; Berger et al., 2019; Weyand et al., 2000). To better understand the value of a performing biomarker and its impact on disease management, a more detailed knowledge of the molecular mechanisms embedded in the pathogenesis of vasculitis is crucial. Specifically, while the role of adaptive immunity has been largely elucidated, innate immunity players are increasingly peeping out, suggesting the need of their in-depth study in the context of vascular inflammation (Ciccia et al., 2017; Akiyama et al., 2021)*.*

When the vasculitic process occurs, the “immune privileged status” of the arterial walls is disrupted. Inflammatory cells penetrate through the adventitia vasa vasorum to the inner layers. As a result, the release of chemoattractants, the expression of adhesion molecules and the recruitment of immune cells enhance the systemic inflammatory response, predisposing to tissue damage and remodeling (Weyand and Goronzy, 2013; Weyand et al., 2019). In addition, it is worth mentioning that the deregulation of the inflammatory response may reside in altered epigenetic mechanisms that control gene expression through DNA methylation, histone post-translational modifications and noncoding RNA, such as micro-RNA (miRNA). (Coit et al., 2018). In this regard, within the temporal artery of GCA patients, DNA methylation changes occur resulting in final hypomethylation of genes involved in TCR/CD28 signaling and calcineurin (Ca)/NFAT-regulatory pathways. NFAT is a transcription factor that regulates the expression of cytokines, including IFNγ and TNF, and the expression of CD40 ligand (Coit et al., 2016). Thus, hypomethylation of cytokine genes for IFNγ, IL-6, TNF, CCR7, and CCL18 would associate with their hyperexpression in the vascular infiltrate. Moreover, miRNAs implicated in the regulation of the inflammatory response were found hyperexpressed not only in T cells, but also in innate cells, such as macrophages and DCs (miR-146a, miR-155, and miR-21) infiltrating the inflamed temporal arteries of GCA patients (Croci et al., 2016).

The two most common large vessel vasculitis, GCA and Takayasu arteritis (TAK), share most of the mechanisms underlying the dysregulation of innate immunity and the main cellular players (Mirault et al., 2017). However, the analysis of arterial samples of TAK remain more limited compared with GCA, preventing a more comprehensive view of its immunopathogenesis. Both GCA and TAK are defined by granulomatous inflammation of the vessel wall as a response to a loss of tolerance and the establishment of a maladaptive immune response (Pugh et al., 2022). In terms of clinical spectrum, the pattern of arterial involvement may differ between GCA and TAK. In addition, unlike TAK, the incidence of GCA increases with age, suggesting that the aging process may influence the development of the disease (Mohan et al., 2011). Two potential concomitant mechanisms may play a role in the increased risk of GCA with age. The first is immunosenescence, characterized by a decline in naïve T and Treg cells, production of pro-inflammatory cytokines, shifts in the distribution of CD4 and CD8 cells, and contraction of T-cell receptor diversity (Goronzy et al., 2007). The second is the age-related remodeling of the vascular wall, defined by the decrease in number and function of VSMCs, degeneration of the media, calcium deposition and thickening of the intima resulting in loss of elasticity and increased stiffness (Lee and Oh, 2010).

In this review, we will discuss the current knowledge on the dysfunctional innate immune responses underlying the pathogenesis of GCA and their implications to explore useful biomarkers associated with improved diagnosis, monitoring, prognosis and therapy (Table 1).

**TABLE 1:** Molecular and cellular biomarkers related to innate immunity and wall resident cells in GCA.

	Correlated biomarkers	References
Macrophages	VEGF	Croci et al. (2016)
	IL-33	Cid et al. (2006)
	YKL-40	Lee and Oh, (2010)
	PDGF	Esen et al. (2021)
	sCD163	Coit et al. (2018)
	angiopoietin-2	Mirault et al. (2017)
	MMP-9	Mirault et al. (2017)
	MMP-2	Mirault et al. (2017)
	calprotectin	Goronzy et al. (2007)
	osteopontin	Coit et al. (2018)
Dendritic cells	TLR 7, 3, 9	Eisenbarth, (2019)
Mast cells	VEGF	Salvarani et al. (2012)
	ST2	
	Spatial location on arterial wall	
Neutrophils	S100A12	Pryshchep et al. (2008)
	CD16^hi^AnxA1^hi^CD62L^lo^CD11b^hi^/CD16^bright^CD62L^dim^CD11b^bright^	Krupa et al. (2002)
	LDN (CD10^lo^)	Han et al. (2008)
	IL-8	Daxini et al. (2018)
ECs	ET-1/ET_B_	Nakae et al. (2006); Mäyränpää et al. (2008)
	MMP-9	Foell et al. (2004)
	sICAM-1	Pillay et al. (2012); Nadkarni et al. (2014)
	p19 subunit of IL-23	Wang et al. (2020)
	IL-32 and IL-33	Akiyama et al. (2019); Sun et al. (2022)
	Anti-ECs Ab	Keshari et al. (2012); Lihui et al. (2020)
	Jagged-1	Weyand and Goronzy (2013); Michailidou et al. (2020)
VSMCs	NGF, BDNF, NT-3, P75^NTR^, TrkB receptor	Dimitrijevic et al. (2010)
	ET-1	Lu et al. (2006)
	IL-32 and IL-33	Ruetten and Thiemermann, (1997)

VEGF, vascular endothelial growth factor; IL-33, interleukin-33; PDGF, platelet-derived growth factor; sCD163, soluble CD163; MMP-9,-2, matrix metalloproteinase-9,-2; TLR, 7,3,9, toll-like receptor 7,3,9; ST2: IL-33, receptor; LDN, low density neutrophils; IL-8, interleukin-8; sICAM-1, soluble intercellular adhesion molecule-1; ET-1, endotelin-1; ET_B_, endotelin-1 B receptor; IL-32, interleukin-32; Anti-ECs Ab, anti-endothelial cells antibodies; NGF, nerve growth factor; BDNF, brain-derived neurotrophic factor; NT-3, neurotrophin-3; P75^NTR^, P75 receptor; TrkB, tropomyosin receptor kinase B receptor.

## Innate immunity and related biomarkers in GCA

### Macrophages

In GCA, macrophages are the central drivers of vessel inflammation and, together with T lymphocytes, participate in granuloma formation as the hallmark of disease (Esen et al., 2021). Monocytes use different chemotaxis pathways to enter tissues: classical monocytes depend mainly on the CCR2-CCL2 axis, while nonclassical monocytes depend on the CX3CR1-CX3CL1 axis. It has been shown that most macrophages in the temporal artery biopsies of GCA patients resemble nonclassical monocytes with expression of CD16 and CX3CR1. In addition, high expression of CCL2 by VSMCs elicited by the inflammatory milieu has been reported in GCA (Cid et al., 2006). In the early stages, they mediate systemic inflammatory responses through the production of pro-inflammatory cytokines, IL-6, IL-1 and tumor necrosis factor (TNF)-α, amplifying the inflammatory cascade and giving rise to the systemic symptoms that characterize the clinical spectrum. On the other hand, macrophages induce neoangiogenesis by the secretion of vascular endothelial growth factor (VEGF), IL-33 and YKL-40, and tissue remodeling mediated by platelet-derived growth factor (PDGF) (Kaiser et al., 1998; Samson et al., 2017; van Sleen et al., 2019; van Sleen et al., 2021). Indeed, PDGF leads to VSMCs proliferation and migration to the intima with subsequent lumen obstruction (Nissinen and Kähäri, 2014). Furthermore, their capability of tissue damage is promoted by the production of matrix metalloproteinases (MMPs), especially MMP-2 and MMP-9, and reactive oxygen species (ROS). As a marker of disease, MMPs are implicated in fragmentation of the internal elastic lamina (Rodríguez-Pla et al., 2005; Fukui et al., 2016). In light of these observations, macrophages-derived serum markers may prove useful for disease targeting. In this regard, soluble CD163, YKL-40, angiopoietin-2, IL-33, VEGF, MMP-9, calprotectin and osteopontin were increased in the serum of GCA patients (van Sleen et al., 2019) and high levels of angiopoietin-2, ESR together with low levels of MMP-3 at baseline correlated with the finding of vasculitis in patients with concomitant PMR (Fukui et al., 2016; van Sleen et al., 2020). Moreover, GCA monocytes produced high amounts of MMP-9, -2, and -7 transcripts, whereas MMP-1, -3, -8, -10 and -12 transcripts were not different in GCA and healthy controls (HC) monocytes (Watanabe et al., 2018a).

Beyond their potential diagnostic role, macrophages-derived serum markers may predict disease course. Specifically, high levels of serum VEGF and angiopoietin-1 at baseline were associated with a short time to glucocorticoid (GC)-free remission, while high levels of serum YKL-40 at baseline were associated with a long time to GC-free remission. High levels of angiopoietin-2 were predictive of a worse disease course and anticipated relapse during treatment, while MMP-2 was negatively associated with GCA relapse (van Sleen et al., 2019). Understanding macrophage heterogeneity in both phenotype and function in vasculitic lesions may suggest a new perspective for GCA patients’ stratification. Macrophages in the tunica media are characterized by tissue-destructive capabilities that rely on MMP-9 production and ROS release. In contrast, macrophages producing PDGF and VEGF are critically involved in wall remodeling and intimal hyperplasia. Recently, further cell subtyping in GCA identified the spatial distinction of CD206^+^/YKL-40^+^/MMP-9^+^ macrophages and FRβ^+^/CD206^-^ macrophages related to tissue destruction and intimal hyperplasia, respectively. CD206^+^/YKL-40^+^/MMP-9^+^ macrophages were located in the media and along sites of elastic lamina degradation. In contrast, FRβ^+^ macrophages were found mainly in the adventitia and intima where they promoted fibroblast proliferation. Granulocyte-Macrophage Colony-Stimulating Factor (GM-CSF) and Macrophage Colony-Stimulating Factor (M-CSF) are soluble mediators able to impact macrophage heterogeneity, inducing overexpression of CD206 and FRβ, respectively. Thus, the spatial gradient of GM-CSF and M-CSF in the inflamed vessel wall would underlie macrophage polarization, which is strongly influenced by the surrounding microenvironment (Jiemy et al., 2020; van Sleen et al., 2021). In addition, macrophages stimulated with M-CSF, rather than GM-CSF, secrete high levels of PDGF, which again confirms their role in promoting fibroblast proliferation (Esen et al., 2021).

The suitability of macrophages from a diagnostic point of view has also been investigated in the development of radiotracers. Among them, [^18^F]NOS, a radiopharmaceutical targeting iNOS, has been successfully used in pulmonary inflammation with abundant infiltration of iNOS^+^ macrophages (van der Geest et al., 2021). Thus, the presence of CD68^+^iNOS^+^ macrophages in the inflamed arteries of GCA patients suggests its potential use in GCA imaging. New candidate radiotracers targeting macrophage subsets in GCA include [^18^F]fluoro-PEG-folate (targeting FRβ), [^68^Ga]NOTA-anti-MMR-sdAb (targeting CD206), [^68^Ga]-labeled anti-CD163-antibody (targeting CD63), and [^68^Ga]DOTA-TCTP- 1 (targeting MMP-2/9) (Eichendorff et al., 2015; Stellingwerff et al., 2015; Kiugel et al., 2018; Moisio et al., 2020; Verweij et al., 2020). Macrophages are confirmed as crucial cells at every stage of the vascular inflammatory process. Therefore, future research on the role of their products in vasculitis lesions could pave the way for their more standardized and appropriate use in the comprehensive management of GCA patients.

### Dendritic cells

DCs are specialized antigen-presenting cells that work as a potent T-cell activator, thereby acting as a bridge between the innate and the adaptive immune response. Human arteries possess their own tissue-resident DCs, the so-called vasDCs, located at the adventitia-media junction. In GCA, TLR-stimulated vasDCs break self-tolerance and promote abnormal T-cell activation with subsequent retention of T cells within the vessel wall (Ma-Krupa et al., 2004; Goronzy et al., 2008; Eisenbarth, 2019). This process involves a dual mechanism: a more pronounced co-stimulatory signal, *via* the CD28^−^CD86 pathway, and a concomitant deficiency in the co-inhibitory signal mediated by the programmed cell death protein (PD)-1/PD-ligand (PD-L)1 immune checkpoint (Zhang et al., 2017; Weyand et al., 2018). This leads to hyperactivation of T cells and production of interferon (IFN)-γ, IL-17 and IL-21 with enhancement of the vasculitic process. Concordantly, even in mice the immature state of arterial DCs could be easily overcome through the reduction of PD-L1 inhibitory expression in response to LPS administration. This resulted in the proliferation of vascular T cells *in situ* and differentiation toward effector cells (Sun et al., 2022).

In addition to DCs, macrophages from GCA patients also show decreased PD-L1 expression, distinguishing them from macrophages from patients with coronary artery disease (CAD), which are characterized by high PD-L1 expression instead. Interestingly, glucose concentration was critical for increasing PD-L1 expression in CAD macrophages but failed to overcome the PD-L1 deficiency typical of GCA macrophages, suggesting a potential connection between glucose metabolic commitment and functional differentiation of macrophages (Watanabe et al., 2018b). Thus, modulation of PD-L1 expression is a shared feature in myeloid cells and appears to play a key role in GCA. Recent advances in cancer immunotherapy have driven the availability of anti-PD1 or anti-PD-L1 therapy to restore anti-tumor T-cell responses. Confirming the substantial role of co-inhibitory signaling deficiency in GCA, several case reports have confirmed that patients treated with checkpoint inhibitors are at risk for therapy-induced vasculitis (Daxini et al., 2018).

Adventitia DCs appear to be early pilots of the vasculitic process *via* TLR activation. TLRs act as a first line of defense, as pattern recognition receptors recognizing pathogen-associated molecular patterns (PAMPs) or danger-associated molecular patterns (DAMPs), and participate in shaping both innate and adaptive immune responses. Either surface TLRs (1, 2, 4, 5, 6, and 10) or intracellular TLRs (3, 7, 8 and 9), upon stimulation, trigger signaling cascades that ultimately culminate in the activation of nuclear factor kappa-light-chain-enhancer of activated B cells (NF-kB) (De Nardo, 2015; Jiménez-Dalmaroni et al., 2016). Moreover, the key role of TLR-mediated DC activation in establishing vascular inflammation could further strengthen the hypothesis of the infectious trigger in the pathogenesis of GCA. In this view, infection derived PAMPs may initiate TLR activation and thus trigger the onset of GCA. However, to date, there is no robust evidence about any definitive causative microorganism (Salvarani et al., 2012). Aberrant expression of TLRs has been demonstrated in peripheral blood mononuclear cells (PBMCs) from patients with GCA, emerging as a useful potential biomarker. Increased expression of TLR7 has been shown on PBMCs in both patients with PMR and patients with GCA. TLR3 was overexpressed on both T and B cells in active GCA, while TLR9 only in B cells. No differences were found for the expression of TLRs 2, 4, 5, 6 and 8 in PBMCs. In addition, GCA patients show altered TLR functions, as evidenced by the decreased TLR7 response during active disease and subsequent normalization during remission, despite its increase in PBMCs (Álvarez Rodríguez et al., 2011). Expression of TLRs differs significantly between vascular territories; TLRs 2 and 4 are ubiquitously expressed in medium- and large-sized vessels, being highly expressed in temporal arteries where they act as potent stimulants for DCs (Pryshchep et al., 2008). Activation of TLR subtypes involves stimulation of different chemokine pathways. Notably, TLR4-mediated stimulation of DCs increases chemokine (C-C motif) ligand 20 (CCL20) production, inducing recruitment of CCL20-responsive chemokine receptor (CCR)6^+^ T cells that dominate vasculitic infiltrates. Once activated, DCs express a number of chemokines, including CCL 18, CCL 19, and CCL 21. Specifically, CCL 19 and CCL 21 are involved in the maturation of DCs and induce T cell responses (Marsland et al., 2005) while CCL 18 contributes to the recruitment of naive T cells (Krupa et al., 2002). Activated DCs also release cytokines such as IL-6, IL-18, IL-23, IL-32, and IL-33 that contribute to the proinflammatory milieu boosting vascular and extravascular GCA features (Han et al., 2008; Sibilano et al., 2014). Taken together, the above evidence emphasized the important contribution to GCA of DCs and their signaling pathways, not only as players in the breakdown of the immune privilege of the vessel wall, but also in the characterization of the vasculitic process.

### Mast cells

Mast cells have been recently evidenced as active cells in several immunological responses linked to autoimmunity and allergy. Once activated, mast cells secrete a wide range of molecules including VEGF (Sibilano et al., 2014), the most important growth factor for chaotic neo-angiogenesis observed in GCA. VEGF strongly affects ECs and VSMCs proliferation sustaining the vascular remodeling that follows inflammatory arterial damage. In this regard, mast cells can play an important role in GCA pathogenesis. Indeed, their activation could be related to the presence of IL-33, a cytokine highly expressed at tissue level in inflamed arteries that may push VEGF release *via* the binding of ST2 receptor, highly expressed on GCA derived mast cells (Ciccia et al., 2013). Beside VEGF, mast cells produce other important inflammatory mediators with a renowned role in GCA, such as TNF-α, IL-6, IL-13, monocyte chemoattractant protein (MCP)-1, MCP-3, and macrophage inflammatory protein (MIP)-1α.

However, the role of mast cells can be far more complex, including potential immunoregulatory functions. In asthma, despite a potential proinflammatory signal derived from the engagement of ST2, it has been shown that chronic exposure to IL-33 induces a hyporesponsiveness in mast cells, raising the intriguing possibility that IL-33 might actually have a protective rather than a causative role in chronic inflammation settings (Jung et al., 2013). In addition, mast cells seem responsible for Treg suppression/regulation *via* IL-9 stimulation, another key cytokine in GCA small vessel vasculitis and transmural inflammation (Ciccia et al., 2015).

Cell-cell interaction and the IL-9 pathway may participate in orchestrating peripheral tolerance at inflammatory sites. By the way, the exact role of the partnership between Treg and mast cells is still far from being fully understood in GCA and may support a protective role of mast cell in the course of disease (Lu et al., 2006). To support a more proinflammatory role of mast cells, a study published in 2008 described the presence of mast cells in the neointima of GCA temporal arteries. Histologically, a close relationship between mast cells, new-generated vessels and T cells was depicted, confirming a potential role of mast cells in the process leading to vascular injury and T cell activation, as previously reported (Nakae et al., 2006). Interestingly, in control biopsies, mast cells were only found at adventitial level and not in the media or intima, making this cells subset a possible tissue biomarker specific of GCA (Mäyränpää et al., 2008).

The geographical mapping and the functional commitment of mast cells may account for their double-sided behaviour with a not univocal role in GCA pathogenesis.

### Neutrophils

Neutrophils are not usually considered key players in GCA pathogenesis because of their paucity in arterial lesions. However, some studies have highlighted their presence in vasa vasorum and small vessels surrounding temporal arteries (Foell et al., 2004). In the small vessels within the adventitial layer neutrophils can promote inflammation through the production of S100 proteins that are believed to directly activate ECs. Specifically, S100A12 is produced by neutrophils and its serum levels are increased in GCA patients compared to controls. In 2018, the levels of such protein were confirmed to be elevated during active disease and performed similarly to ESR and CRP as indexes of disease activity in GCA (Springer et al., 2018).

The observation of persistent neutrophilia in GCA, even occurring under steroid treatment, suggested a putative role for neutrophils in GCA development and relapse. To test this hypothesis the immunophenotyping of neutrophils at different timepoints of steroid treatment (48 h, 1–4 and 24 weeks) was performed. At 48 h from treatment introduction neutrophils still showed a markedly active phenotype (CD16^hi^AnxA1^hi^CD62L^lo^CD11b^hi^), while after 1 week a clear shift towards an hyporesponsive phenotype (CD16^hi^AnxA1^hi^CD62L^lo^CD11b^lo^), unable to interact with ECs, was observed. In contrast, neutrophils at 24 weeks gain back their activated signature and their aberrant ability to adhere to ECs. In addition, a specific subset of neutrophils, identified as CD16^bright^CD62L^dim^CD11b^bright^, was found to have T-suppressing function (Pillay et al., 2012) and was specifically decreased in GCA patients under treatment at week 24, suggesting a possible mechanism of escape that may account for GCA relapse (Nadkarni et al., 2014). A very recent paper, demonstrated the presence of immature low density neutrophils (CD10^lo^) in both blood and temporal biopsies from GCA patients. In particular, immature neutrophils seem resistant to apoptosis and able to aberrantly interact with platelets. In GCA temporal arteries these cells were preferentially located in the lumen and in the adventitial layer, suggesting that they may not be directly involved in granuloma’s formation but they could likely be initiators of the elastic lamina disruption. Functionally, immature neutrophils were shown to be active producers of ROS. ROS enhance neutrophil degranulation and support marked endothelial inflammation with consequent arterial wall damage. The observation that immature neutrophils were not found in healthy controls, coupled with their reduction after steroid treatment, suggests a potential role of this subpopulation as a cellular specific marker of GCA (Wang et al., 2020).

To sum up, the characterization of neutrophils subsets can be useful to identify early GCA, potentially re-emergent disease and the development of treatment resistance. In the proinflammatory milieu characterizing GCA the activation of neutrophils may be related to a series of key cytokines, such as IL-17 and IL-9. IL-17 is a major soluble mediator of disease as it bursts granulopoiesis, neutrophil migration and release of chemokines from phagocytes. Regarding IL-9, the overexpression of IL-9 receptor in neutrophils was evidenced, especially in transmural and small vessel vasculitis GCA histologic pathotypes. Upon IL-9 stimulation neutrophils release IL-8 whose levels are strongly increased in GCA, accounting for a functional immunological link between IL-9 receptor expression and neutrophil activity in GCA arteries (Ciccia et al., 2015).

Finally, a fascinating relationship has been described between neutrophils forming extracellular traps (NETs) and MMP-9 induction in granulomatosis with polyangiitis (GPA) (Akiyama et al., 2019). GPA and GCA share common features and the common pathway of tissue damage related to MMP-9 deserve to be further elucidated investigating a possible role of NETosis in GCA (Akiyama et al., 2021). So far, a clear demonstration of NETosis activation in GCA is lacking. However NETosis inducing cytokines, specifically IL-1β, are highly hyper-expressed in GCA inflamed arteries and *in vitro* essays have demonstrated that IL-1β induces NET production as well as IL-8 and TNF-α can activate the pathway driven by NAPDH oxidase with consequent release of ROS and NET formation (Keshari et al., 2012; Lihui et al., 2020). More studies focusing on NETosis in GCA are required to clarify its role as a new pathological mechanism and to help the identification of novel biomarkers of disease (Michailidou et al., 2020).

## Resident cells in the artery wall and related biomarkers in GCA

The vessel wall constitutes the microenvironment where vasculitis occurs. In heathy condition the wall structure can be considered an immune-privileged tissue where no aberrant inflammatory response takes place. The three-layer architecture acts as a shield against unwanted inflammatory triggers (Akiyama et al., 2021). However, in GCA, the tissue damage starts with the disruption of the arterial barrier through the digestion of the extracellular matrix driven by MMP-9 (Cui et al., 2017). Wall components, such as ECs and VSMCs are actively implied in the pathogenesis of wall damage and contribute to orchestrate the complex immune response characterizing GCA. In addition, these cells are massively involved in the wall remodeling that follows the early stage of disease with consequent aberrant neo-angiogenesis and growth of neointima (Rodríguez-Pla et al., 2005). The final result is arterial occlusion and ischemia.

### Endothelial cells

ECs represent the interface between tissues and blood at both the intimal layer of medium and large vessels as well as at vasa vasorum and neovessels level. ECs influence the homeostasis of the vessel structure regulating hemostasis, vasomotion, angiogenesis and inflammation (Samson et al., 2017). In particular, ECs express MHC molecules and intercellular adhesion molecules that drive cell-cell interaction and immune cells translocation from blood flow to inflammatory sites. In GCA, ECs display an hyperactivated phenotype, as demonstrated by the increase in endothelin-1 (ET-1) and endothelin-B receptor (ET_B_) expression in temporal artery biopsies (Dimitrijevic et al., 2010). ET-1 is a potent vasoactive peptide able to modulate the sensitivity of arterial wall to several vasoconstrictive molecules, such as serotonin, noradrenaline and angiotensin II. ET-1 proatherogenic and proinflammatory role has first been established in atherosclerosis and then described in GCA (Pache et al., 2002; Ivey et al., 2008), depicting an overlapping scenario between the two diseases. Specifically for GCA, ET-1 and ET_B_ expression positively correlates with the magnitude of systemic inflammation as mirrored by the concomitant marked increase in ESR and CRP. This observation is paired with the evidence that ET-1 drives NF-kB and TNF-α induction, shaping a strong proinflammatory milieu and not simply inducing vasoconstriction and proliferation of ECs (Ruetten and Thiemermann, 1997; Wilson et al., 2001). As a result of the NF-kB activation an increase in MMP-2 and MMP-9 is evidenced (Felx et al., 2006), further enhancing matrix degradation in GCA. In addition, ET-1 is responsible for the discharge of several cytokines and chemokines, such as IL-8 and MCP-1 from macrophages, and for the production of ROS, all factors deeply affecting wall tissue damage (Stankova et al., 1996).

Tissue alterations are determined by immune cells infiltration into the arterial wall. ECs mediate the recruitment of leukocytes *via* the expression of constitutive adhesion molecules, namely platelet endothelial cell adhesion molecule (PECAM)-1, intercellular adhesion molecule (ICAM)-1, ICAM-2 and P-selectin, and inducible adhesion molecules, such as E-selectin and vascular cell adhesion molecule (VCAM). The overexpression of adhesion molecules is specifically identified among ECs of inflamed adventitial microvessels and neovessels and drives inflammatory infiltrates development and maintenance. Intriguingly, the intensity of inducible adhesion molecule expression and the concentration of soluble ICAM-1 correlate with the severity of disease activity (Coll-Vinent et al., 1999; Cid et al., 2000). The expression of adhesion molecules is boosted by IL-23p19 that was found increased in ECs from GCA patients. In particular, under proinflammatory conditions, ECs seem able to synthetize the p19 subunit of IL-23. Intracellular p19 binds the cytokine receptor subunit gp130 activating the gp130-dependent activation of signal transducer and activator of transcription (STAT)-3 signalling cascade with the consequent upregulation of VCAM-1 and ICAM-1 (Espígol-Frigolé et al., 2016). ECs belonging to neovessels scattered through the inflammatory infiltrates can also directly produce proinflammatory cytokines, such as IL-32 and IL-33, fostering the aberrant immune response observed in GCA (Ciccia et al., 2011; Ciccia et al., 2015). Interestingly, the expression of ST2, IL-33 receptor, is upregulated in GCA ECs. Its engagement activates the STAT6 cascade promoting angiogenesis, vascular leakage and tissue infiltration by immune cells. In addition IL-33, in parallel with other inflammatory molecules, such as TNF, oxidised low-density lipoprotein (ox-LDL), IL-1β and CRP (Seelentag et al., 1987; Rajavashisth et al., 1990; Devaraj et al., 2009), boosts the production of GM-CSF and M-CSF from ECs, further contributing to inflammatory activation of the arterial wall (Montanari et al., 2016).

To add complexity to the role of ECs in GCA it has been pointed out that these cells may serve as a source of autoantigens potentially triggering autoimmunity in GCA. Anti-ECs antibodies targeting laminin A/C, vinculin, annexin V and voltage-dependent anion-selective channel protein 2 were detected in GCA sera. Up to date, the pathogenic role of anti-ECs antibodies has not been validated and they are not used as disease markers in clinical practice (Régent et al., 2011; Kuret et al., 2018). Finally, ECs are the principal culprits of the vascular remodeling that deeply depends on neo-angiogenesis promoted by VEGF. VEGF enhances the proliferation of ECs and upregulates Jagged-1 expression on ECs surface. Jagged-1 is the ligand of NOTCH-1, that is highly expressed by T cells in GCA, explaining the intimate interaction between ECs and effector T cells (Weyand and Goronzy, 2013; Simons et al., 2016; Wen et al., 2017). The study of ET-1, adhesion molecules, IL-23, VEGF/NOTCH1 and GM-CSF pathways related to ECs are all possible future biomarkers of disease. Importantly, their blockade may reveal new therapeutic targets that deserve to be further evaluated in GCA to pioneer more selective treatment options for patients.

### Vascular smooth muscle cells

VSMCs are the most represented cell subset of the arterial wall. They mainly reside in the media and promote contraction and tissue healing. The inflammatory background of GCA pushes VSMCs to improperly migrate to the intima, where they differentiate, together with fibroblasts (Parreau et al., 2021), into myofibroblast able to produce matrix proteins. Both migration and new-tissue formation contribute to the final vascular occlusion of the artery’s lumen with consequent block of blood flow (Piggott et al., 2009).

VSMCs can release a wide range of growth factors, such as PDGF, VEGF, TGF-β and neurotrophins, such as nerve growth factor (NGF) and brain-derived neurotrophic factor (BDNF) that activate VSCMs themselves in an autocrine loop (Ly et al., 2014). The presence of neurotrophins can help in identifying patients as, at tissue level, the immunostaining for neurotrophins is more intense in GCA versus controls and, particularly, neurotrophin-3 (NT-3) and P75 receptor (P75^NTR^) are only detected in GCA temporal artery specimens, serving as possible biomarker of disease (Régent et al., 2017). Strikingly, the tropomyosin receptor kinase (Trk) B, that binds BDNF, was almost exclusively expressed in GCA patients who experienced cranial ischemic events, such as amaurosis, permanent visual loss or stroke. At peripheral level, in the same study, NGF serum concentration was found significantly higher in GCA patients. The anomalous wound healing response promoted by VSMCs is possible in presence of adequate angiogenetic activity which is necessary to support VSCMs nutritional supply. In this regard, the release of MMP-9 and MMP-2 from VSCMs facilitates the destruction of the media and the internal elastic lamina favouring the formation of new vessels, normally restricted to the adventitia, at media and intima level. The migration and proliferation of VSMCs is further enhanced by the production of ET-1 and the overexpression of ET-1 receptor, both A and B, was found in GCA VSMCs (Simons et al., 2016). Moreover, ET-1 levels are increased even at peripheral level and are associated with risk of retinal ischemic events (Pache et al., 2002; Lozano et al., 2010). The contribution of VSMCs to initiate and perpetrate vascular damage in GCA is underlined by the active production of cytokines from these cells. In particular a strong expression of IL-32, and to a lesser extent of IL-33, was evidenced in VSMCs from GCA temporal artery samples. VSMCs are exposed to oxidative stress in GCA and, as a result, can increase the release of “alarmins”, such as IL-33, favouring inflammation (Rittner et al., 1999).

VSMCs can be definitely considered central cells in the development of arterial lesions during GCA and several mechanisms behind their pathological behaviour can be addressed as new biomarkers and treatment targets in the next future.

### Fibroblasts

In the process leading to intimal hyperplasia and fibrosis fibroblasts seem to play an important role. These cells reside in the adventitial level (Stenmark et al., 2013), but upon pro-inflammatory stimulation, can migrate to the intima and polarize their phenotype to myofibroblast able to produce collagen and growth factors, such as TGF-β1 (Shi et al., 1996). TGF-β1can further activate, in an autocrine loop, fibroblasts to increase their migration to the inner part of the arterial wall and perpetrate wall damage and architectural alteration of the three layer vessel structure. One recent paper analysed the distribution of fibroblasts across the temporal artery wall. CD90^+^ cells, identified as fibroblasts, were found increased in the adventitial and in the intima layers derived from GCA patients respect to controls (From the American Association of Neurological Surgeons et al., 2018). These cells penetrate the destroyed media to move towards the intima, probably by the stimulation of PDGF-BB and NAPDH oxidase-4, as evidenced in the vascular remodeling of other disease, such as hypertension (Barman et al., 2014; Dutzmann et al., 2017). Moreover, fibroblasts co-expressed high level of alpha-smooth muscle actin, marker of activated myofibroblasts, in GCA specimens, especially in the adventitia. This observation strengthens the plasticity of fibroblasts, usually considered quiescent cells, when exposed to an inflammatory microenvironment, as described in arterial inflammatory disease (Matsubara et al., 2020).

Fibroblasts not only contribute to the transmural anatomical alterations of the temporal artery but are also active producers of IL-6 in GCA granulomas (Emilie et al., 1994). IL-6 release supports the strong inflammatory status typical of GCA, especially in the active phases of disease. More research is needed to deeply explore fibroblasts role in GCA development and the correlation between fibroblastic infiltration and the clinical-biological features of disease to possibly use these cells as markers of disease severity.

## Innate immune cells as therapeutic targets in GCA

Glucocorticoids (GCs) remain the cornerstone therapy in GCA, although their efficacy is mainly to suppress extravascular GCA and acute phase response, while they do not sufficiently treat vascular GCA (Régent and Mouthon, 2022). IL-6 is a cytokine involved in the development of acute phase responses, and its levels are elevated in the serum and inflamed artery of patients with GCA. These observations justify the efficacy of tocilizumab, an IL-6 receptor antagonist, in preventing recurrence and reducing the dose of GCs (Villiger et al., 2016). Tocilizumab is currently the only bDMARD approved for GCA. Greater understanding of the immune signaling networks underlying vascular inflammation has led to the identification of a variety of potential therapeutic targets in addition to IL-6, and several clinical trials have recently been completed or are currently underway (Macaluso et al., 2022). In recent years, the efficacy of JAK inhibitors in the treatment of rheumatic diseases has been highlighted. Contextually, the demonstration of increased JAK-STAT pathway activity in vascular lesions of patients with GCA and the involvement of molecules such as IL-6, type I IFN and GM-CSF, all of which utilize the JAK-STAT pathway, make the efficacy of JAK inhibitors against GCA highly likely (Watanabe and Hashimoto, 2022). Recently, baricitinib, an inhibitor of JAK1 and JAK2, was reported to be effective against relapsed GCA in an open-label prospective study (Koster et al., 2022). In addition, a clinical trial of another JAK inhibitor for GCA (NCT03725202, upadacitinib) is ongoing. GM-CSF is a multifunctional cytokine implicated in the pathogenesis of GCA through modulation of DCs, CD4^+^ T lymphocytes, and macrophages (Wicks and Roberts, 2016). Blockade of the GM-CSF receptor in cultured temporal arteries resulted in decreased expression of markers in these cell populations and decreased transcription of genes associated with Th1 and Th17 immune responses (e.g., interferon-γ and interleukin-6) (Corbera-Bellalta et al., 2022). In a phase 2, randomized, double-blind, placebo-controlled trial, Mavrilimumab, a monoclonal antibody that blocks GM-CSF signaling, administered with a 26-weeks prednisone taper, significantly reduced the risk of flare and improved sustained remission rates compared with placebo plus a 26-weeks prednisone taper in patients with GCA (Cid et al., 2022). Other potential therapeutic approaches of interfering with disease-relevant molecules could involve blocking agents of MMP-9, a key molecule in elastic membrane disruption and particularly important in GCA aortitis complications. In a preclinical model, treatment with an antibody blocking MMP-9 activity was sufficient to arrest vasculitis and prevent vessel wall remodeling (Watanabe et al., 2018a). Furthermore, given the critical function of VEGF as an endothelial cell regulator, the use of anti-angiogenic interventions could have benefits in controlling the vascular inflammatory process (Wen et al., 2017).

## Conclusion

**FIGURE 1:**
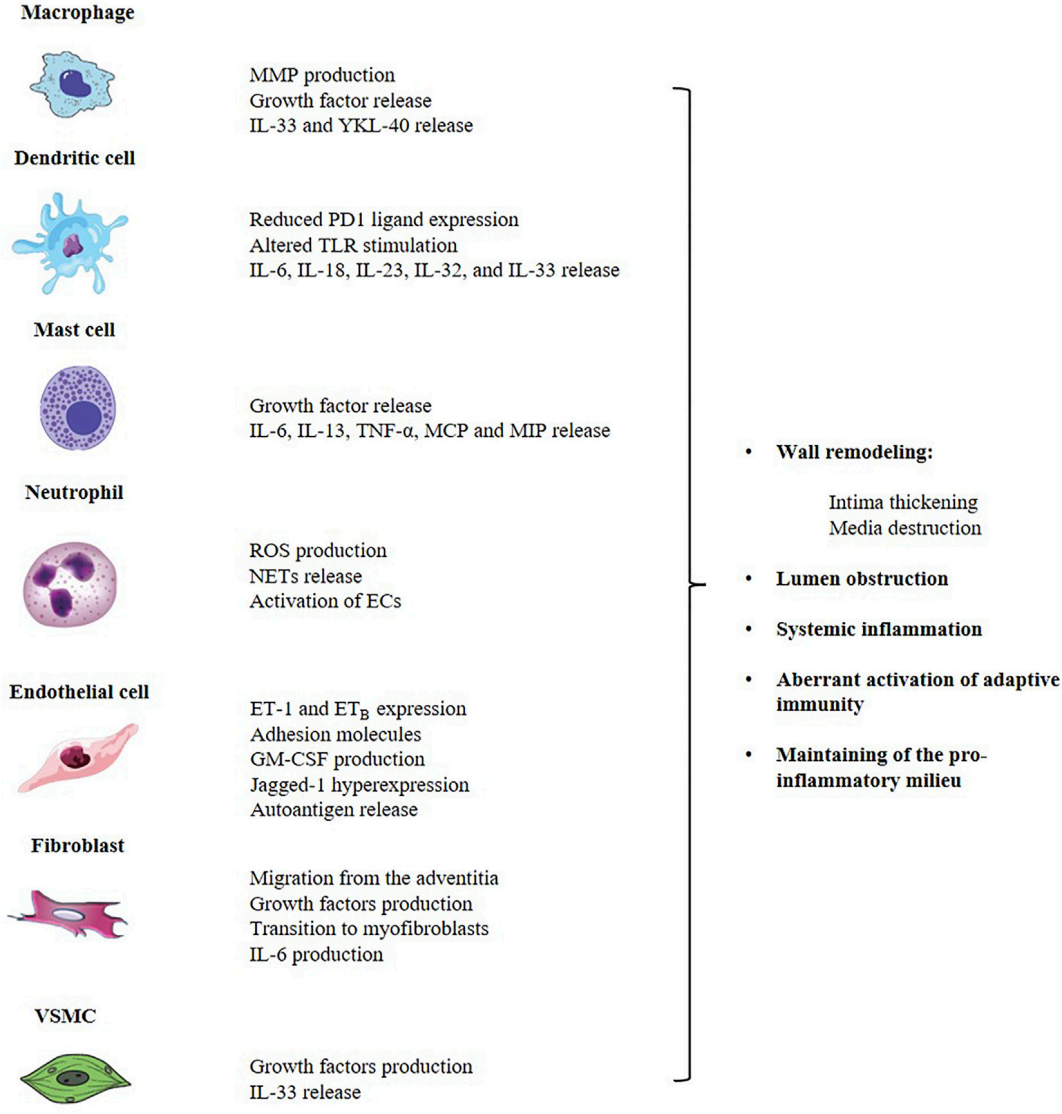
The innate immune system contributes to the pathogenesis of GCA through the aberrant activation of the adaptive system and the direct production of a complex network of cytokines, chemokines and growth factors that are responsible for the arterial wall remodeling and the induction of a pro-inflammatory status, at both local and systemic level. Main players involved in GCA are arterial wall macrophages, DCs, neutrophils, mast cells and components of the vessel wall, namely ECs, fibroblasts and VSMCs. GCA, giant cell arteritis; ECs, endothelial cells; DCs, dendritic cells; MMP, matrix-metalloproteinase; IL, interleukin; MCP, monocyte chemoattractant protein; MIP, macrophage inflammatory protein; TLR, toll-like receptor; ROS, reactive oxygen species; NETs, neutrophil extracellular traps; ET-1, endotelin-1; ET_B_, endotelin-1 B receptor; VSMC, vascular smooth muscular cells.

GCA is both an autoimmune and autoinflammatory condition affecting large and medium arteries with potentially life-threatening complications. The disruption of self-tolerance affects both arms of the immune system. However, the contribution of the innate system to the genesis of the infiltrate could be crucial through the interaction with T cell themselves and direct production of effector cytokines, chemokines and growth factors. Beside arterial wall macrophages, literature data describe an important role for DCs, neutrophils, mast cells and components of the vessel wall, namely ECs and VSMCs (Figure 1). All cells contribute to inflammation and structural damage *via* the production of MMP-9, VEGF, PDGF, IL-33, IL-9, adhesion molecules and vasoactive mediators, through an active and coordinated network in which the endothelium serves as a critical player in facilitating leukocyte trafficking in the arterial wall and the development of key signaling pathways of vascular inflammation. Up to date, no accurate biomarker is available to diagnose and monitor GCA course and available treatments for GCA have important limitations. Thus, there is an urgent need for new plasma and imaging biomarkers that can both identify active disease, in its vascular and extravascular component, and monitor response to treatment, in order to grant a better prognostic stratification in GCA. Furthermore, as a result of advances in research into the pathophysiological pathways involved in GCA, several other molecules are currently being evaluated. To conclude, the complexity of GCA pathogenesis reflects the actual challenge for early diagnosis, rapid detection of relapse and personalized treatment that rheumatologists face in everyday clinical practice. A new conceptual approach should definitely include the study of innate immunity pathways in GCA, as they hold promise to provide new strategies for anti-vasculitic immunotherapy and optimal disease assessment.

## Glossary

BDNF: brain-derived neurotrophic factor
C-GCA: cranial GCA
CCL20: chemokine (C-C motif) ligand 20
CCR: chemokine receptor
DAMPs: danger-associated molecular patterns
DCs: dendritic cells
ECs: endothelial cells
ESR: erythrocyte sedimentation rate
ET-1: endothelin-1
ET_B_: endothelin-B receptor
GCA: giant cell arteritis
GC: glucocorticoid
GM-CSF: Granulocyte-Macrophage Colony-Stimulating Factor
GPA: granulomatosis with polyangiitis
HC: healthy controls
ICAM: intercellular adhesion molecule
IFN: interferon
IL: interleukin
LV-GCA: large-vessel GCA
M-CSF: Macrophage Colony- Stimulating Factor
MCP: monocyte chemoattractant protein
MIP: macrophage inflammatory protein
MMPs: matrix metalloproteinases
NF-kB: nuclear factor kappa-light-chain-enhancer of activated B cells
NETs: neutrophils forming extracellular traps
NGF: nerve growth factor
NT-3: neurotrophin-3
PAMPs: pathogen-associated molecular patterns
PBMCs: peripheral blood mononuclear cells
PCR: C-reactive protein
PD: programmed cell death protein
PD-L: PD-ligand
PDGF: platelet-derived growth factor
PECAM: platelet endothelial cell adhesion molecule
P75^NTR^: P75 receptor
PMR: polymyalgia rheumatica
ROS: reactive oxygen species
SAA: serum amyloid A
STAT3: signal transducer and activator of transcription 3
TNF: tumor necrosis factor
TLR: toll-like receptor
Trk: tropomyosin receptor kinase
VCAM: vascular cell adhesion molecule
VEGF: vascular endothelial growth factor
VSMCs: vascular smooth muscle cells
